# Supplementary material for: Current evidences of the efficacy of mosquito mass-trapping interventions to reduce Aedes aegypti and Aedes albopictus populations and Aedes-borne virus transmission
Source: PLoS Negl Trop Dis. 2023 Mar 6;17(3):e0011153. doi: 10.1371/journal.pntd.0011153 (PMC10032496; doi:10.1371/journal.pntd.0011153)
Supplement: Attachment — Submitted filename: 2022-08-31_ResponseReviewers_fv.docx [file pntd.0011153.s001.docx]

**Reviewer's Responses to Questions**

**Title**

**Reviewer #1: It is not general “Aedes” but Aedes invasive species.**

**It is not general “Aedes” but Aedes invasive species. Possible title could be: Current evidence on the effectiveness of mosquito mass-trapping interventions to control invasive Aedes populations and to reduce the transmission of their diseases: A systematic review**

We thank the reviewer for his/her suggestions. We agree with the reviewer that both *Ae. aegypti* and *Ae. albopictus* species are invasive, especially in temperate regions, and we will insist on this information in the text (page 4, line 51). The title “Current evidences of the effectiveness of mosquito mass-trapping interventions to control *Aedes* populations and to reduce the transmission of *Aedes*-borne diseases: A systematic review” has been replaced by “Current evidences of the efficacy of mosquito mass-trapping interventions to reduce *Aedes aegypti* and *Aedes albopictus* populations and *Aedes*-borne transmission”.

**Abstract**

**Reviewer #1: Although authors consider “exclusion criteria” there are no exclusion criteria mentioned in the main text.**

We added the Table 2 to address this relevant comment.

**A more general suggestion is that abstract would highly benefit from some more numerical data to support your main findings.**

We thank the reviewer for his/her suggestion. As we pinpoint in the discussion, at this stage, the heterogeneity of the indicators used to assess trap efficacy (number of host-seeking females, number of gravid females, proportion of positive containers, viral infection rate in female mosquitoes or serological studies in residents) is a significant limitation to having standardized numerical data and conducting meta-analyses. We have added in the abstract (lines ~32 to 36) “*Our review showed great heterogeneity in the indicators used to assess trap efficacy: e.g. the number of host-seeking females, the number of gravid females, the proportion of positive containers, the viral infection rate in female mosquitoes or serological studies in residents*.”). Therefore, despite this appropriate suggestion, we consider that any numerical data or quantitative indication in the abstract will give an erroneous message at this stage. We add a sentence in the abstract to state that “*More studies with standardized methodology and indicators are urgently needed to provide more accurate estimates of their efficacy*. “ (page 2, lines 38-39).

**Reviewer #3: Abstract**

**-	Specify geographical scope of review**

We added “worldwide” in the abstract (page 2, line 27).

**-	Findings are very poorly presented – insert more detail on range of effect that can be expected from such an intervention. Conclusions: can be more to the point**

See response to reviewer #1. Nowadays and with the available evidences, the current heterogeneity of standardization on the entomological indicator measures and the low sample size of standardized studies (as can be clearly see in Figure 2) makes it difficult to have an accurate effect size. This is stated in the abstract and we decide to be cautious to not under/overestimate the effect. As the evidences for the efficacy of many other *Aedes* (innovative or not) vector control strategies are weak and limited with only particular exceptions, we are convinced of the value of our work contributing to advance and foster the evaluation and incorporation of mass-trapping strategies as a complementary tool in integrated *Aedes* management in particular situations.

**Introduction:**

**-	Not clear why there is a focus on France and the French territories in the introduction – would be better to open up to all endemic areas?**

We thank the reviewer for his/her comment and have clarified in the introduction that the study is worldwide. The reference to France has been removed in order to adopt a more global perspective.

**Introduction**

**Reviewer #1: Authors should give a clear statement and information for invasive mosquitoes (Aedes) and their VBDs.**

We have taken the reviewer's suggestion and added clear information about invasive mosquitoes (*Aedes*) and their VBDs in the introduction: “*Over the past 50 years, the world has seen the emergence and dramatic spread of Aedes-borne arboviral diseases such as dengue, chikungunya and Zika, which are transmitted by the two invasive mosquitoes Aedes aegypti and Ae. albopictus [1]. Diseases caused by these viruses are most prevalent in tropical regions transmitted principally by Ae. aegypti and their incidence are increasing in temperate regions of the world, mainly due to the geographical expansion of Ae. albopictus. Several factors contribute to this risk: globalization of trade and travel, unplanned urbanization, climatic changes, water storage and waste disposal and limitations in the effectiveness of disease or mosquito control strategies [2]*” (page 4, lines 49-56).

**Authors should provide more details on the management of these 2 Aedes. Indeed, it is too complicated, especially in urban areas. Why authors think that mass trapping is a potential method to reduce/control Aedes invasive populations?**

We thank the reviewer for his/her comment. We have added more details in the text on the control of these two *Aedes*, particularly in urban areas. We have also clarified the potential of mass trapping to reduce/control their populations: “*Despite the health, economic and social importance of Aedes-borne diseases and the existence of numerous studies evaluating vector control methods and strategies, the evidence for the public health value of Aedes vector control is unfortunately weak [6].* (page 4, lines 66-69).

*Larval control, and in particular, community-based source reduction and larviciding, is a dominant paradigm for Aedes mosquito control. These methods targeting larvae show moderate efficacy in operational conditions but to maximize their impact on the reduction of virus transmission, is important to reduce Aedes density and longevity [7]. Although the use of insecticides remains the predominant strategy for controlling adult female Aedes in case of epidemics, there are limited entomological and epidemiological evidence on the efficacy of ultralow volume (ULV) space spraying with some evidence of efficacy in some settings for Indoor Residual Spraying (IRS) or insecticide-treating materials [6]. It is important to note that authorities and citizens worldwide have a growing aversion to the use of insecticides, due to their limitations in efficacy, the emergence of insecticide resistance and their environmental and health impacts. Therefore, the development and evaluation of non-insecticidal tools targeting different fractions of the target population (host-seeking females, blood-fed females, etc.) should be fostered. New approaches—such as the Sterile Insect Technique (SIT), Incompatible Insect Technique (IIT), genetically modified mosquitoes, removal trapping, and spatial repellents— are being evaluated in the field and are providing relevant entomological/epidemiological data on their efficacy. Recently, there is entomological evidence for mass deployment of lethal oviposition traps to reduce Aedes mosquito density [Johnson, 2017]. Mass trapping can be a low-cost, community-based, and sustainable complementary engagement approach, attractive to complement other tools that can be combined and selected locally within an integrated Aedes management strategy. There is no silver bullet for Aedes-borne diseases control, and the most practical and productive path forward is to strengthen the evidence base so that, when appropriate, an effective ‘toolbox’ can be deployed in an integrated manner taking into account the local situation and available resources [6].* “ (page 4, lines 70 to page 5, line 92).

**Reviewer #1: Lines 60-68. Why do the authors make a specific reference on the case of France, while this is a global study? Maybe the study would benefit from a more global perspective and/or European. In Europe aegypti is not established so, discus the 2 species separately (e.g. for Ae. albopictus also check Bellini, et al 2020. Travel medicine and infectious disease, 35, p.101691.).**

The reference to France has been deleted in order to adopt a more global perspective. Some paragraphs (see before) have been added in order to clarify why mass trapping can be useful as part of the plethora of tools that can be combined for integrated *Aedes* management.

**Reviewer #1: Lines 95-97. More references could be cited on the linkages of aedes and urban environments (especially systematic reviews).**

We have taken the reviewer's suggestion and added the following systematic review in the references: Kolimenakis A et al. (2021). The role of urbanisation in the spread of Aedes mosquitoes and the diseases they transmit—A systematic review. PLoS Negl Trop Dis 15(9): e0009631. https://doi.org/10.1371/journal. pntd.0009631

Similarly, we added the following sentence in the introduction: “*Females of Ae. albopictus and Ae. aegypti have a specific behavior of laying eggs in man-made containers, especially in urbans environments. Several studies have shown an association between urbanization and the distribution and density of these both Aedes vectors (Kolimenakis A et al., 2021*) (page 6, lines 114-115).

**To support the objective of the study, authors should revise the introduction in a way that would highly benefit from further evidence on previous studies and how this study steps to them. In addition, the explanation of the scope of this review is very short and could be expanded to 3-4 bullet points.**

The introduction has been subsequently revised to address this comment and the scope of the review has been expanded “Therefore, the objective of this work was to perform a scoping review of the existing scientific literature to assess the efficacy of mosquito trap-based interventions in controlling *Aedes* population densities and the diseases they transmit, informing control intervention as well as building proof of concept to guide future studies.” (Page 6, lines 118-122).

**Methods**

**Reviewer #1: According to authors only 2 databases were used from their searches namely Pubmed and Scopus. The authors should explain in more details why they did not utilize other databases for their searches.**

In the methodology section (page 7, lines 134-135), we have specified that Scopus and Pubmed are two major databases in scientific literature (Scopus is a multidisciplinary database and PubMed is mainly focused on life sciences database). Other databases exist but were not solicited, such as WoS, which is not easily accessible, and Google scholar, which cannot perform precise searches.

**Reviewer #1: Eligibility criteria. This is not clear to me. No reference is made on any exclusion criteria used for this systematic review while criteria 4 and 6 are both on the study type (maybe consider merging?).**

We thank the reviewer for this comment. We have added a table (Table 2) specifying the exclusion criteria.

**Reviewer #1: Lines 155-156. The authors should further expand on whether any tool was used for the quality assessment of the included studies.**

All authors discussed the quality of the included studies. A spreadsheet was completed to discuss the various possible biases related to these trials (design study, coverage, local environment, duration…) for each study.

**Reviewer #2: The authors present a systematic review following PRISMA guidelines of published evidence on the effectiveness of mosquito mass-trapping interventions to control Aedes populations and to reduce the transmission of Aedes-borne diseases.**

**Authors discuss how data can be helpful to advance our understanding of the topic under study and its public health relevance. Limitations are mentioned.**

We thank the referee for this comment.

**Reviewer #3: - This paper is more a scoping review than a systematic review**

We agree with the reviewer and have replaced "systematic review" with "scoping review" throughout the text.

**Reviewer #3: Field studies:  In the title, effectiveness is mentioned, but in some of the papers it looks more as an efficacy study – evaluating effect in 3 houses is not an effectiveness study. Hence, a better definition of ‘field studies’ is needed – suggestion to call it ‘community studies’**

We agree with the reviewer and have replaced ‘field studies’ by ‘community and field studies’. We have replaced ‘effectiveness’ by ‘efficacy’.

**Reviewer #3: Observational studies: some of the observational studies included were not ‘analytical study that quantify the effect of an outcome’, such as reference 22 and 29. Coverage is a process indicator and not an outcome of effectiveness indicator**

We agree with the reviewer. We have replaced ‘effectiveness’ by ‘efficacy’ in the text. We agree that coverage is not an outcome of effectiveness but an indicator of the quality of implementation. The two references by Acevedo et al (2016) and Ritchie et al (2009) presenting only results on the public acceptability of mass trapping have been removed from Table 3.

**Reviewer #3: Data extraction and analysis: very good to have seasonality and coverage included as variables extracted**

We thank the referee for this comment.

--------------------
**Results**

**Reviewer #1: Table 2. Authors should present in a clearer way how the 21 included studies are cited in the summary tables 2 and 3 Please consider adding the name of authors instead of reference and restructuring the table for a clearer reference of each included study. Why there is no reference of the main findings of each included study, apart from the reduction achieved? This is important in order to more clearly present the policy perspectives of this study.**

The names of the authors have been added in the Tables. Main findings of each included study have been completed.

**Reviewer #3: -In the introduction, there is a clear presentation of 2 categories of traps – but this clear difference is disappearing in the methods/results section (especially the latter). Suggestion to improve structure of paper, following these two categories: give summarized result per categorie and afterwards give the stratified result per sub-categorie. Now it reads as 5 different result-stories, where the reader looses the oversight.
A restructure into the following could improve clarity:**

- **Resuming effectiveness estimates on entomological indicators (differentiate if used as a sole measures or as part of a combined strategy)**
- **Resuming effectiveness estimates on epidemiological parameters (differentiate if used as a sole measures or as part of a combined strategy)**
- **Resuming factors influencing the effectiveness of the tools**

The structure of the article has been partially modified in order to take into account the reviewer’s proposition. We have 4 different result-stories with 2 species of *Aedes* and 2 categories of traps (see Figure 2).

**Reviewer #3: Another general comment on the results-section: often the indicators in the control area are not mentioned, this is for dengue/Aedes very important, as we know that there is a high variability over seasons and years and often interferes with measurement of effectiveness. Best to always report on intervention and control area, or if there is no control area, to state it, so that the readers knows how to interpret the findings. This information need to be added to the table (effect in control area/or use relative effect/’reduction achieved’). -**

According to the reviewer’s suggestion, the text has been modified as the title of the last column of the table, which has been completed, in order to report lack of information in studies and results of studies without effect.

**Reviewer #3: Lines 184 and 187: contradiction: ‘there is an at least 40% decrease’ <-> ‘observed impact was low’: what do authors mean? Best to keep the facts in the results section and the interpretation in the discussions session.-**

We thank the reviewer for his/her suggestion, the commentary ‘observed impact was low’ has been suppressed.

**Reviewer #3: Line 202: it reads as if in this study the LO were used to evaluate the intervention, but were not part of the control efforts – please clarify and if so, this study needs to be taken out -**

The LO were used to be part of the control efforts. Indeed, “*The use of locally manufactured lethal ovitraps was the method used to reduce the adult vector population of Ae. aegypti that was not eliminated in the immature stages by all other vector control methods”.*

**Reviewer #3: Line 206-207: clarify this sentence – is this important information? It reads like a story and not as results of a research. -**

We agree with the reviewer, the sentence has been rephrased.

**Reviewer #3: Line 227 - line 232: in one sentence the effect is the same in the four sites, and further down authors state that the site with ‘integrated interventions’ is better than the other sites – please clarify -**

According to the reviewer’s suggestion, the text has been clarified. Indeed, the results obtained in this study do not indicate a specific efficacy of LOs in reducing *Ae. aegypti* populations. Indeed, the results showed a decrease in *Ae. aegypti* abundance after the interventions but in a similar way in the four neighborhoods studied (LOs, Bti or LOs + Bti or control). The lack of significant differences suggests that the integrated control strategy with social mobilization and participation of the population in the elimination of breeding sites as implemented in all sites offers better results than the exclusive use of one of the available tools (LOs, Bti or LOs + Bti).

**Reviewer #3: Line 245-247: were the traps used as a control measure or as an evaluation tool? - Chapter on ‘autocidal gravid ovitraps’ – reads too much as a story and not as a scientific article. For the reader it’s difficult to appraise scientific evidence from it. –**

A sentence has been added to precise that the traps were used for both control and evaluation measures: ‘*Mosquitoes collected by the BG-Sentinel set in intervention area were also used as a population measure with* *regular ovitraps (dry season) or SOs (wet season). In the untreated control area, 15 BGS traps and regular ovitraps (dry season) or SOs (wet season) were also used to assess adult population.*’ (page 15, lines 264-267).

Moreover, it has been specified that acceptability was measured as the failure of residents to remove LOs and BLOs set in their yards and that traps were used as a control measure.

**Reviewer #3: Line 271, line 280: reduction or relative reduction, taking into account the oscillation of parameters in control area? -Insert something on comparability of areas pre-intervention**

We thank the reviewer for his/her comment. It has been specified that it is a relative reduction, compared to the control.

**Reviewer #3: Lines 372 – 376: opinion of authors or evidence from study?**

According to the reviewer’s suggestion, we decided to delete this paragraph, as it is not based on solid evidence.

**Reviewer #3: The title of the last column of the table 2: ‘reduction achieved’ – suggestion to make this ‘effectiveness measured’. I have also the impression that if there is no reduction found, that the authors don’t give a lot of information on effectiveness. This is biasing the readers with drawing too much attention to the studies with effects (and not reporting as good the studies without effect) – example study 32, study 18. Mind also to give the precise figures of the main indicator of each study.**

Based on the reviewer’s suggestion, the title of the last column has been modified and the table has been completed, in order to report the lack of information in some studies and results of the studies without effect.

**Information on public acceptability could be presented in a separate table.**

We agree with the referee. The two references by Acevedo et al (2016) and Ritchie et al (2009) presenting only results on the public acceptability of mass trapping have been removed from Table 3. The public acceptability of vector control methods remains a major parameter to be taken into account in these trials. The information from these two publications is therefore well presented and discussed in the text. We had retained "sociological indicators" in the selection criteria, which explains why these two studies were selected. Indeed, we aimed to select studies of mass trapping against *Aedes* that included entomological and sociological indicators. However, this is not the case. The two studies with sociological indicators do not have entomological indicators. None of the entomological efficacy studies of mass trapping that we retained included sociological indicators.

**Reviewer #3: Line 391: ‘no effect over entire period’ – but there was a significant effect in winter time – does this means that there was an adverse effect in summer? (as the average is zero effect, with a significant effect in one period).**

In this study, there is no significant effect for the whole period but only for a shorter one, when mosquito density is low (in summer). It is not possible to conclude that there is an adverse effect for such a short period and for only one study. The trapping limit is reached in case of high mosquito density (see coverage question).

Following the reviewer’s request, we have modified the text accordingly.

**Reviewer #3: Lines 393-401: what is opinion of the authors and what is evidence?**

Following the reviewer’s request, we have modified the text accordingly. We have removed the interpretations of the authors of these studies to keep only the facts (results) in the results section of the review for each article. Some of these relevant interpretations have been included in the discussion of the review. Overall, the results have been clarified so that the scientific evidence can be assessed for each study and between studies.

**Reviewer #3: Lines 402 – 431: consider taking out of this review as it is not an effectiveness study, but efficacy studies in single (or 3) house(s). Also LO were tested in single houses and this was not included in this review.**

Indeed, our review is more on efficacy than effectiveness. According to this comment, we have modified the title and the text of the article. As we read the paper, we understand that ovitraps are used as a trap to measure the secondary outcome ie. the egg abundance of *Ae. albopictus*, and not as an intervention method. Results of the reduction of the abundance of *Aedes albopictus* eggs has been added in the article.

--------------------

--------------------

**Conclusions**

**Reviewer #1: I strongly suggest to authors to revise the discussion section to expand specifically on the added value of the study on previous conducted studies.  Additionally, the discussion would highly benefit from few bullet points of the main outcomes of the study. Authors should consider to include reference(s) on the main limitations of this study and any actions to mitigate them.**

**Moreover, is important to discuss further why (or not) this method should be adopted by stakeholders against Aedes invasive mosquitoes.**

**The last comment (lines 570-576) is very important for mosquito control programs. Authors should consider connecting ovitraps with QC (there are 2 relevant references about evaluation of methods from one of the authors; Michaelakis et al. 2021. International Journal of Environmental Research and Public Health, 18(7), p.3478 & Abramides et al. 2011. Transactions of the Royal Society of Tropical Medicine and Hygiene, 105(5), pp.281-288).**

Following the reviewer’s request, we have modified the text accordingly. (see discussion, page 36-37).

**Reviewer #2: General conclusions are well supported by the data presented: “This review highlights a knowledge gap in demonstrating the effectiveness of mass trapping of mosquitoes in reducing viral transmission and disease. As a result, further large-scale Phase III cluster randomized controlled trials conducted in endemic areas and including epidemiological outcomes are needed to establish scientific evidence for the reduction of viral transmission risk by mass trapping targeting gravid and/or host-seeking female mosquitoes.”**

We fully agree with that citation. This is our conclusion

**However, some “conclusions” are incomprehensible. Particularly “All of these considerations point to the need for an international website on vector control trials. This website would be an online repository where teams conducting vector control trials could document the study design, type of intervention, geographic area targeted, objectives, endpoints, estimated duration, and sample size, and where contact information for the research team could be provided. This approach is used by most credible research teams working on human clinical trials and is becoming a regulatory requirement in many countries. Such a benchmark could be used by scientists around the world to assess other research being conducted elsewhere – where to fill in the gaps, where not to duplicate efforts, and where to create synergies. The rigor of these trials can be assessed by the quality of these protocols. Such labelling would also make it more difficult for commercial trapping systems to claim that their products have been properly tested and are effective if they have not. False claims of efficacy are a public health problem because people trust the wrong protection methods and are at greater risk of being infected.”**

**What is the proposal? An “new” international website on vector control trials? Is there any one now? Do the authors know VCAG?**

**If we don’t have the new website, what is being currently done is not credible? and not fulfilling regulatory requirements?**

**Labelling and claiming for commercial trapping systems is (and should) be addressed in each country; and indeed, should be honest and demonstrable.**

Following the reviewer’s request, we have modified the text accordingly. We have removed all the paragraph about the website and only mentioned existing platforms, such as VCAG (see discussion, page 37).

**Reviewer #3: The discussion talks too much about evidence in vague terms, such as ‘potential evidence’, ‘could increase’,** …

Following the reviewer’s request, we have modified the text in order to be more accurate in the terms used.

**Reviewer #3: Line 448: report on the incidence/immunity to chikungunya prior to intervention in both areas, as this could influence effect**

Information on incidence/immunity to CHIKV have been specified when available in the articles, and are already mentioned in the corresponding tables.

**Reviewer #3: Line 455: what is ‘aggressive density’?**

We thank the referee for this suggestion. Thererfore, “aggressive density” has been replaced by “Average number of female *Ae. albopictus”.*

**Reviewer #3: There is a lot of repetition in the discussion – it would improve if more concise and to the point**

The reviewer’s comment has been taken into account; we have modified the text accordingly (see new version of the discussion).

**Reviewer #3: Line 482: what do authors mean with ‘exclusion areas to vector control’?**

“Exclusion areas to vector control” has been replaced by “existence of exclusion zones for insecticide treatments” (page 34, line 516)

**Reviewer #3: Line 495: Social mobilization is normally added to an intervention strategy and is not an intervention on its own/it is a co-strategy to achieve mechanical elimination/control of breeding sites. Rephrase sentence.-**

Integrated vector control includes social mobilization in most of the cases, right. The sentence has been reformulated in this way.

**Reviewer #3: Line 507: the potential difference between urban and rural is an interesting focus, and was not sufficiently included in the review -**

Most mass trapping trials against *Aedes* vector populations have been performed in urban areas, we consider that we cannot speculate on a difference between urban and rural without enough representative studies.

**Reviewer #3: Line 545 and following: why setting up a new repository if clintrials.gov already exists and contains several of the vector control studies. Perhaps an option making submission compulsory to the existing repositories?**

We thank the reviewer for this proposition. Our suggestion now is to systematize a set of information (such as type of intervention, targeted geographical area…) in any review and to make sure that existing platforms take all these data into account.

**Reviewer #3: Line 555-558: too much speculation for a scientific paper?**

Following the reviewer’s request, we have modified the text accordingly. We have removed all the paragraph about the website and only mentioned existing platforms, such as VCAG (see page 36).

--------------------

**Reviewer #3: The subject of this paper is interesting and important for decision making. However, it reads more as a story than as a systematic review. This could be improved upon by keeping the facts (results) in the results section and the interpretation in the discussions session, now there is a mix, which makes it hard to know what evidence is existing.**

Our article is more a scoping review than a systematic review. We have removed the interpretations of the authors of these studies to keep only the facts (results) in the results section of the review for each article.
