# Supplementary material for: Current evidences of the efficacy of mosquito mass-trapping interventions to reduce Aedes aegypti and Aedes albopictus populations and Aedes-borne virus transmission
Source: PLoS Negl Trop Dis. 2023 Mar 6;17(3):e0011153. doi: 10.1371/journal.pntd.0011153 (PMC10032496; doi:10.1371/journal.pntd.0011153)
Supplement: Attachment — Submitted filename: 2022-11-28_ResponseReviewers_fv.docx [file pntd.0011153.s002.docx]

**Reviewer's Responses to Questions**

Key Review Criteria Required for Acceptance? As you describe the new analyses required for acceptance, please consider the following:

**Methods**

-Are the objectives of the study clearly articulated with a clear testable hypothesis stated?

-Is the study design appropriate to address the stated objectives?

-Is the population clearly described and appropriate for the hypothesis being tested?

-Is the sample size sufficient to ensure adequate power to address the hypothesis being tested?

-Were correct statistical analysis used to support conclusions?

-Are there concerns about ethical or regulatory requirements being met?

**Reviewer #1**: All the objectives and the design of the study are clearly explained.

**Reviewer #2**: (No Response)

**Reviewer #3**:

**o	Please insert how quality/bias was evaluated in the papers selected. We agree with the reviewer, as it is the quality that has been evaluated.** The term bias was replaced by quality of the paper. Page 10, line 186-189 “*All authors discussed the quality of the included studies. A spreadsheet was conceived to collect and evaluate information on study design, coverage area, local environment and duration of the interventions in order to assess the quality of the selected studies and their alignment with the objectives of our analysis*”.

o	**Table 1 and Table 2: rephrase the definition of ‘comparator’ – what is now written in the box, is the description of study type (except between brackets)**

We defined a comparator as an “Intervention site compared to a control site (in the case of contemporary treated/control areas) or baseline data obtained at the same site (in the case of pre/post compairisons)”. (Page 8, table 1)

**o	Table 2: study type: observational study is both an inclusion as an exclusion criterion🡪** Observational study is an inclusion criterion.

--------------------

**Results**

-Does the analysis presented match the analysis plan?

-Are the results clearly and completely presented?

-Are the figures (Tables, Images) of sufficient quality for clarity?

**Reviewer #1**: All the results are clearly presented.

**Reviewer #2**: (No Response)

**Reviewer #3: Effect/efficacy/effectiveness – the authors are mixing these concepts all over the text. Revise these concepts: for example - table 3 last column: use ‘effect’ in the title if there is a combination of efficacy and effectiveness studies (which is the case in this scoping review). Most of the studies are effectiveness studies, as they are applying tools in real life conditions, a few are efficacy studies as they are evaluating tools in few houses under very controlled circumstances. See also line 541, 532, 438..**

We thank the reviewer for this comment. “Efficacy” has been replace by “effect” in Table 3 and Table 4 and in the text, lines 438, 532 and 541.

--------------------

**Conclusions**

-Are the conclusions supported by the data presented?

-Are the limitations of analysis clearly described?

-Do the authors discuss how these data can be helpful to advance our understanding of the topic under study?

-Is public health relevance addressed?

**Reviewer #1**: All the conclusions are presented and public health relevance is clearly addressed.

**Reviewer #2**: (No Response)

**Reviewer #3: See the remarks on the abstract and the synopsis - looks like conclusions stay very vague**

We made an effort to be as veridical as possible based on the tangible data available. The limitations of the conclusions are due to the limitation of the available -epidemiological and entomological - data assessing the effectiveness of mass-trapping and the lack of standarization that complicates a solid data analysis.

**lines 450-452 : what is an experimental bias?**

Experimental bias is an overestimate or underestimate of the effect of an intervention. We change the sentence to be more pertinent “*However, the strength of evidence of these results remains moderate because they may be subject to bias* [36].” (page 28, lines 459-461).

--------------------

**Editorial and Data Presentation Modifications?**

Use this section for editorial suggestions as well as relatively minor modifications of existing data that would enhance clarity. If the only modifications needed are minor and/or editorial, you may wish to recommend “Minor Revision” or “Accept”.

**Reviewer #1**: Accept

**Reviewer #2**: (No Response)

**Reviewer #3**: (No Response)

--------------------

**Summary and General Comments**

Use this section to provide overall comments, discuss strengths/weaknesses of the study, novelty, significance, general execution and scholarship. You may also include additional comments for the author, including concerns about dual publication, research ethics, or publication ethics. If requesting major revision, please articulate the new experiments that are needed.

**Reviewer #1: Authors addressed all my concerns and suggestions. I strongly suggest them to highlight more their aspect for evaluation and monitoring of the mosquito control programs and support the last two paragraphs, in discussion, with relevant references.**

We thank the reviewer for his/her suggestion. We have added in the discussion : *“Finally, we emphasize that disease control programs must be evaluated and monitored. Evaluation of these programs in terms of public health is straightforward but should be carried out in conjunction with an economic evaluation to measure the cost effectiveness and cost-benefit of different methods (traps, larviciding, adulticiding, etc.) and vector control strategies. A****n integral and constant evaluation allows for a better understanding and grants the adjustment of measures and their adaptation to local conditions to expand the impact of these programs.*** *Together, these data can provide a body of evidence-based information that can guide cost-effective and sustainable vector control strategies, thereby reducing the disease burden and economic impact of vector-borne diseases.* ***The pertinent scrutiny of these elements allow decision makers to consider the appropriate approach and ensures the success of an integral strategy****”. (page 33, lines 644-654).*

**Reviewer #2**: The authors have followed and made the changes and suggestions of the reviewers in a satisfactory manner.

**Reviewer #3**: Most of the comments of first review were adequately addressed and paper improved consistently. However, here are still some remarks on abstract, synopsis and introduction

-	Abstract: line 36 – ‘various studies support the efficacy….’. This is really too vague for an abstract. Alternatives: XX% of studies demonstrated a reduction of entomological indicators; or; another way could be to report on the quality of the studies included in the review – if you score them ‘low quality’, hence it is OK that you can’t say anything in the abstract.

We consider that any numerical data or quantitative indication in the abstract will give an erroneous message at this stage.

**-	Synopsis: ‘this review provides some evidences …’ – as in abstract, reads like authors are not convinced of the effect of these tools – to do with quality of papers included? – rephrase and be clear (as in abstract)**

The sentence has been replaced by “*This review support the efficacy of mass trapping in combination with classical integrated vector control on the reduction in mosquito vector populations within several weeks of deployment*”. (Page 4, lines 52-55)

**-	Introduction:**

**o	Line 71: rephrase ‘… with significant economic impacts.’**

The sentence has been replaced by “*Therefore, the control of Aedes-borne diseases becomes a public health and a socio-economic burden*”.

o	**Line 82: insert reference**

A reference (Roiz *et al*., 2018) has been added

o	**Line 84-88: no reference made to the systematic reviews done on larval control methods and adult control methods**

We added larval control methods and some references. We referred to the paper from Roiz *et al*., 2018 “*Integrated Aedes management for the control of Aedes-borne diseases*” provided a table summarizing vector control tools for *Aedes* mosquito control (Table 2) and an other table summarizing the strength of the evidence supporting vector control (S2 Table 2). We added some other references : Buhler et al. (2019), Maoz et al. (2017), Esu et al. (2010) and Boyce et al. (2013).

o	Line 95-96: … providing relevant entomological/epidemiological data on their efficacy’ – what is meant by this? Please add a reference

- “ *New approaches—such as the Sterile Insect Technique (SIT), Incompatible Insect Technique (IIT), genetically modified mosquitoes, trapping, and spatial repellents—are being evaluated in the field and are providing relevant entomological/epidemiological data on their efficacy*” has been replaced by “*Significant progress has been made and new techniques such as the Sterile Insect Technique (SIT), Incompatible Insect Technique (IIT), genetically modified mosquitoes, trapping, and spatial repellents are currently in different stage of development and have already provided the scientific community with an array of data on their efficacy”* (Douchet *et al*. 2021).

o	Line 97: Authors rightfully refer to the review on traps of Johnson (reference 8) – but would be good to mention what this paper adds to the reference 8 (in introduction or discussion) – many of the conclusions are the same

We have added the following sentence : “*Our work complements and updates the review by Johnson et al. [12] and differs from it in two aspects. Firstly, from a methodological point of view, we have chosen a systematic review, which facilitates the updating of evidence as new results are available. Secondly, we have broadened the scope of mass trapping from gravid traps to host-seeking traps, with a view to considering several tools that can be included in an integrated pest management strategy*”.

o	Lines 98-99: rephrase: ‘… complementary…complement …combined…’

The sentence has been replaced by “*Mass trapping can be a low-cost, community-based, and sustainable engagement approach, attractive to complement other tools that can be selected locally within an integrated Aedes management strategy”.*
